# Exploring microplastic impact on whole blood clotting dynamics utilizing thromboelastography

**DOI:** 10.3389/fpubh.2023.1215817

**Published:** 2023-07-13

**Authors:** Alexei Christodoulides, Abigail Hall, Nathan J. Alves

**Affiliations:** ^1^Department of Emergency Medicine, Indiana University School of Medicine, Indianapolis, IN, United States; ^2^Weldon School of Biomedical Engineering, Purdue University, West Lafayette, IN, United States

**Keywords:** thromboelastography, microplastics, coagulation, thrombosis, TEG, polystyrene, nanoplastics

## Abstract

This study investigates the influence of microplastics on blood clotting. It addresses the lack of comprehensive research on the effects of microplastic size and surface modification on clotting dynamics in human whole blood. Thromboelastography was used to examine aminated (aPS), carboxylated (cPS), and non-functionalized (nPS) polystyrene particles with sizes of 50, 100, and 500  nm. Results show that cPS consistently activated the clotting cascade, demonstrating increased fibrin polymerization rates, and enhanced clot strength in a size and concentration-dependent manner. nPS had minimal effects on clotting dynamics except for 50  nm particles at the lowest concentration. The clotting effects of aPS (100  nm particles) resembled those of cPS but were diminished in the 500  nm aPS group. These findings emphasize the importance of microplastic surface modification, size, concentration, and surface area on *in-vitro* whole blood clotting dynamics.

## 1. Introduction

Exposure to microplastics – generally agreed to be particles <5 mm in size - in humans remains a significant problem present in society (1). Microplastics can be classified as “primary” or “secondary” depending on if they occur as a byproduct of manufactured plastics or if they are formed by mechanical/photochemical degradation of larger plastics in the environment (2). Routes of exposure to microplastics – whether primary or secondary – in humans can be subdivided into three main categories: dermal, oral, and respiratory. Exposure to these microplastics occurs because of microplastic presence within multiple realms of the environment including food, water, and even the air. Microplastic translocation into the aforementioned “compartments” stems from the heavy reliance of plastics in our daily lives whether it be in packaging materials, cosmetics, medical devices, and paints to name a few. Furthermore, the process of recycling consumer plastics can also be a significant source of environmental microplastics. A 2023 study conducted in the UK demonstrated that plastic recycling facilities can generate huge amounts of microplastic burden in their waste water – estimated to be between 59 and 1,184 tons annually for particles <5 μm (3). Cox et al. estimated that the level of human exposure to microplastics remains around 74,000 to 121,000 particles per person per year, when factoring in both inhalation and ingestion routes (4). Translocation of microplastics, particularly submicron particles (i.e., nanoparticles), into systemic circulation has been documented, and is well-summarized by Lett et al. (5). Of 22 volunteers tested in the Netherlands, 17 had quantifiable levels of microplastics in whole blood samples with polyethylene terephthalate and polystyrene being the most common plastics (6). Of note, the size cutoff in the Leslie et al. study was ≥700 nm particles, thus excluding a large proportion of smaller particles that are known to translocate into the circulatory system.

Provided the tremendous ability of micro/nanoplastics to translocate into the blood stream, a great interest exists in better understanding their physiologic impact, as it pertains to coagulation and hemostasis (7). Additional pursuits of therapeutic micro/nanoparticles has also sparked research in the aforementioned areas as well (8). A multitude of papers, summarized in Lett et al., demonstrate the interest that exists in exploring the impacts of microplastics on coagulation (5). Notably, polystyrene has been the most commonly studied microplastic to date due to its documented prevalence in waste and, therefore, prevalence in human blood samples (9). As a result of the multitude of sizes, surface modifications, and unique functionalization that polystyrene is available in, literature exploring the effects of polystyrene has spanned a broad spectrum of particle types (5). The most common surface modifications of polystyrene explored include non-functionalized polystyrene (nPS), aminated polystyrene (aPS), and carboxylated polystyrene (cPS).

**Figure 1:**
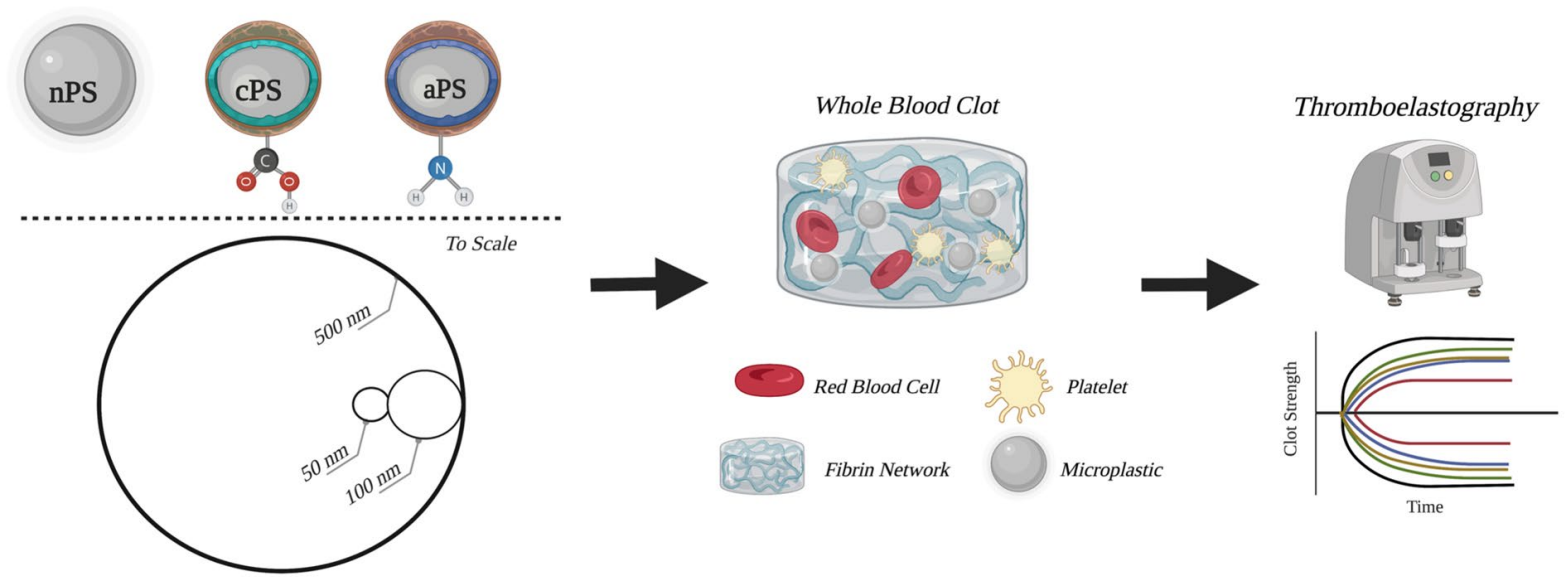
Schematic outline of experimental groups and methods. Circular depictions of the particles are to scale. Figure was created with BioRender.com.

Paralleling the diversity of polystyrene particle subtypes, a wide spectrum of approaches to understanding effects on coagulation has also been undertaken. Systems that have been employed included use of purified coagulation factors, human plasma, computational models, human whole blood, and *in-vivo* animal models of thrombosis (5, 10–15). Presently, there is a lack of consensus within the field regarding correlating particle type, size, surface modification, and shape to physiological impacts in clotting. Thus, additional research to advance the field is necessary. Through our work, we have aimed to help address these gaps by systematically testing a spectrum of polystyrene particle surface modifications, sizes, and particle concentrations; employing the clinical tool of thromboelastography (TEG) to capture the global effects that these plastic particles have on whole blood clotting dynamics (Figure 1). As much of the prior literature focuses on particle impact on discrete activation or inhibition steps within the coagulation system leveraging just a few purified coagulation factors, we aimed to take a more wholistic approach leveraging whole blood impact on bulk coagulation dynamics via TEG analysis.

## 2. Materials and methods

### 2.1. Whole blood collection

Venous whole blood (WB) was collected from healthy volunteers (*n* = 3) by a trained phlebotomist, in accordance with guidelines/methods outlined in our institutionally approved IRB protocol (1610652271). All collection and handling of human specimens has been priorly approved by the IRB at our institution. Blood was collected into 3.2% Sodium-Citrate tubes and immediately pooled into 15 ml tubes for use (BD Vacutainer, Franklin Lakes, NJ). Volunteers reported no recent use of medications that might impact coagulation.

### 2.2. Microplastic particle preparation and storage

Spherical polystyrene (PS) microplastic particles (MP) were purchased from PolyScience (Warrington, PA). For the purposes of this study, three groups of microparticles were explored – aminated PS (aPS), carboxylated PS (cPS), and non-functionalized PS (nPS). Both nPS and cPS were acquired in 500, 100, and 50 nm sizes, while aPS was only manufactured in 500 and 100 nm sizes. None of the particles were fluorophore labeled. All MPs were stored at 4°C in accordance with manufacturer recommendations. MP concentrations in WB utilized included 25, 100, and 250 μg/ml. All calculations were based on the w/v% provided on each MP stock by the manufacturer. Dilutions of 1:10 or 1:5 were done in PBS (10 mM, pH 7.4) prior to the introduction of MPs into the WB samples, and just prior to thromboelastography. Proper mixing via inversion or pipetting was done during each step of the process to account for particle settling. When not in use, MP stocks were stored at 4°C.

### 2.3. Thromboelastography

Prior to initiation of thromboelastography (TEG), 1.5 ml of WB was aliquoted from the stock and brought to room temperature. MPs were introduced into the WB aliquot in accordance with the necessary volume to achieve a desired final concentration, as outlined in section “Microplastic particle preparation and storage.” Of note, a 0 μg/ml condition was also included in our study which involved introducing 75 μl of PBS into the 1.5 ml WB aliquot to control for any dilutional effects in the experimental groups. Daily fresh WB was utilized for each day of the experiment to eliminate the effects on coagulation dynamics that arise from storage of WB (16). TEG was utilized to capture clotting dynamics in all samples. Samples were run in accordance with manufacture-provided protocols for the TEG-5000 instruments (Haemonetics, Boston, MA), with instrument eCalibration performed between each set of samples. TEG clotting parameters of interest included R-time (clotting cascade activation), K-time (time to initial fibrin polymerization), Maximum Amplitude (MA; clot strength), and Angle (rate of fibrin polymerization). Additional TEG parameters collected included Time to Maximum Amplitude, Clotting Index, and G all of which can be found in Supplementary Tables 1−3. For a review of TEG parameter interpretation please reference Willis et al. (17). Of note, the control groups did utilize Kaolin, as outlined in the manufacturer’s protocol. Baseline TEGs for all volunteers were as expected (Supplementary Figure 1). All experimental groups, including the 0 μg/ml group, did not utilize Kaolin to ensure perturbation of clotting dynamics by the MPs could best be captured. All samples were run in quadruplicates. During loading, 340 μl from each sample (outlined in section “Microplastic particle preparation and storage”) was pipetted into a plain TEG cup which contained 20 μl of 12 mM CaCl_2_, samples were gently resuspended twice utilizing a pipette to ensure proper mixing prior to run initiation. All samples were run for 60 min prior to termination to ensure all clotting parameters were fully captured. Note, clot lysis was not observed in any of our samples. All experiments were performed over the course of 3 days, each day was dedicated to testing a MP of a particular size across all concentrations and surface modifications.

### 2.4. Data collection and analysis

All data exported from the TEG instrument was collected and processed in Microsoft Excel (Microsoft, Redmond, WA). Data is presented as mean ± standard deviation unless specified otherwise. Student *t-*tests were utilized to compare between two categorical variables. To account for an increased rate of type I errors that could arise from the multiplicity of pairwise comparisons performed, a Bonferroni correction was employed. Mixed model regressions were performed utilizing SPSS (IBM, Armonk, NY). Significance, for the purposes of this study, were results with a *p*-value ≤0.01. Of note, results with a *p-*value of >0.01 and ≤0.05 are still displayed on all figures for completeness.

## 3. Results

### 3.1. Non-functionalized polystyrene

**Figure 2:**
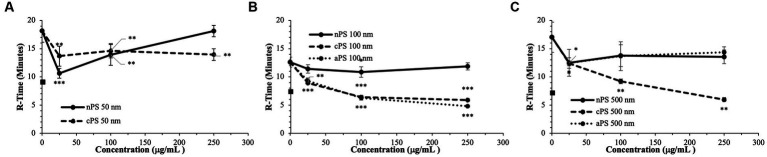
Speed of clotting cascade activation (R-time) split by particle size: 50  nm **(A)**, 100  nm **(B)**, 500  nm **(C)**. Square marker along y-axis represents data point for Kaolin containing samples. Differences in kaolin sample values between days is representative of physiologic differences expected of healthy volunteers. Single asterisk (*) denotes *p*-value ≤ 0.05. Double asterisk (**) denotes *p*-value ≤ 0.01. Triple asterisk (***) denotes *p*-value ≤ 0.001.

**Figure 3:**
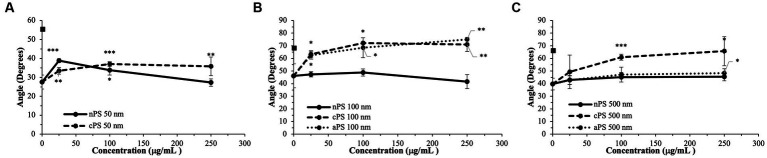
Rate of fibrin deposition (Angle) split by particle size: 50  nm **(A)**, 100  nm **(B)**, 500  nm **(C)**. Square marker along y-axis represents data point for Kaolin containing samples. Differences in kaolin sample values between days is representative of physiologic differences expected of healthy volunteers. Single asterisk (*) denotes *p*-value ≤ 0.05. Double asterisk (**) denotes *p*-value ≤ 0.01. Triple asterisk (***) denotes *p*-value ≤ 0.001.

**Figure 4:**
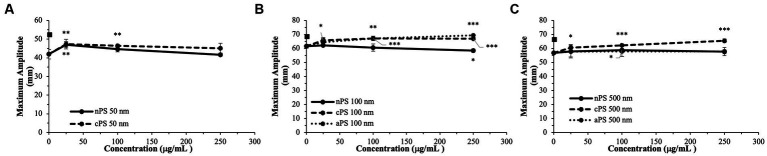
Maximal clot strength (Maximum Amplitude) – a readout of both platelet and fibrin contributions to clot strength - split by particle size: 50  nm **(A)**, 100  nm **(B)**, 500  nm **(C)**. Square marker along y-axis represents data point for Kaolin containing samples. Differences in kaolin sample values between days is representative of physiologic differences expected of healthy volunteers. Single asterisk (*) denotes *p*-value ≤0.05. Double asterisk (**) denotes *p*-value ≤ 0.01. Triple asterisk (***) denotes *p*-value ≤ 0.001.

**Figure 5:**
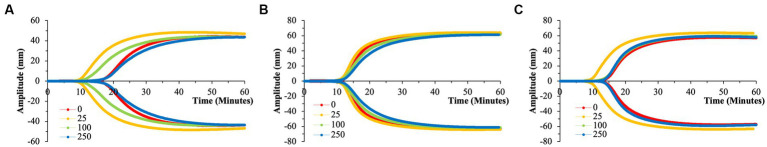
Representative TEG tracings for nPS with color-coded tracings based on the concentration of microparticles utilized. **(A)** 50  nm nPS. **(B)** 100  nm nPS. **(C)** 500  nm nPS. Pronounced effects of the 25 μg/ml group. All tracings pictured were from a single channel (out of the quadruplicate) and are displayed as such for clarity purposes. **(A)** Has a differing *y*-axis scale that is reflective of the variation in clotting dynamics that is expected of healthy volunteers.

The smallest size of nPS tested was 50 nm spheres. Minimal perturbations to the whole blood clotting dynamics – R-time (Figure 2), Angle (Figure 3), and MA (Figure 4) – were witnessed at this size, except at the lowest concentration of 25 μg/ml. A significantly reduced R-time was seen at this concentration – 10.6 ± 0.9 from 18.2 min ± 2.0 (*p*-value < 0.001). This increase in speed of clotting cascade activation nearly approximated the effects of Kaolin on the WB samples’ R-time (9.1 ± 0.3 min). A statistically significant reduction was maintained until the 100 μg/ml group after which any reductions in clot initiation speed were no longer recorded. Of note, Kaolin serves as a positive control in our study as its high surface area coupled with anionic surface charge make it a strong activator of the intrinsic clotting cascade. Exclusion of Kaolin from experimental samples serves to highlight the effects of the plastic microparticles on clotting dynamics. Changes in R-time were paralleled by concomitant significant rises in both MA (*p*-value < 0.01) and rate of fibrin polymerization (*p*-value <0.01) from controls, once again only in the 25 μg/ml groups. However, unlike the changes seen with R-time, they did not approximate the values seen in the Kaolin-containing groups. 500 and 100 nm nPS particles mimicked the 50 nm particle trend of faster clotting cascade activation with the introduction of 25 μg/ml concentrations; however, statistical significance was not achieved at any concentration level. Clotting dynamics for nPS particles at 100 nm and 500 nm sizes remained uniformly unchanged from their respective controls when it came to maximal clot strength and speed of fibrin polymerization. Representative nPS TEG tracings can be observed in Figure 5 with additional TEG parameters seen in Supplementary Table 1.

To draw statistical comparisons between the various nPS particle size groups, standardization had to be performed to account for baseline variation in clotting attributes expected across the healthy volunteers (Supplementary Figure 2). Standardization was accomplished by dividing the respective value from each TEG channel by the respective average of the daily quadruplicate controls (i.e., 0 μg/ml group). Comparisons performed in the aforementioned manner demonstrated the significant and drastic manner by which 50 nm nPS particles were able to influence clotting, most significantly at the 25 μg/ml concentration compared to any other nPS particle size or concentration.

### 3.2. Carboxylated polystyrene

**Figure 6:**
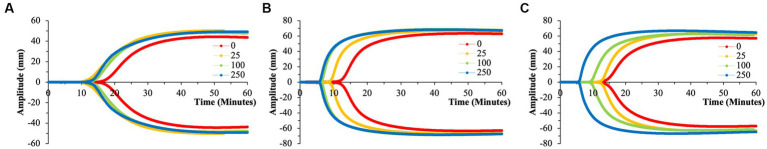
Representative TEG tracings for cPS with color-coded tracings based on the concentration of microparticles utilized. **(A)** 50  nm cPS. **(B)** 100  nm cPS. **(C)** 500  nm cPS. Concentration dependent effects, especially at larger microparticle sizes. All tracings pictured were from a single channel (out of the quadruplicate and are displayed as such for clarity purposes. **(A)** Has a differing y-axis scale that is reflective of the variation in clotting dynamics that is expected of healthy volunteers.

Akin to the nPS particles, cPS particles began to significantly impact the rate of clotting initiation starting at the 50 nm size and 25 μg/ml concentration, with a 23.1% reduction in R-time (Figure 2) and 29.6% reduction in K-time by the 250 μg/ml concentration. In contrast to nPS, these changes were amplified with both increasing concentrations and increasing particle size, such that the largest deviation from baseline was seen with 500 nm particles at 250 μg/ml – 6.0 ± 0.4 versus 17.0 ± 2.7-min R-time (64.7% change, *p*-value < 0.01). Changes in the rate of cascade activation and time to initial fibrin polymerization repeatedly surpassed even the rates of samples initiated with Kaolin in both the 100 and 500 nm cPS groups at concentrations ≥100 μg/ml (Figure 3). Rates of fibrin polymerization mirrored the trends of R-time/K-time in that rises in rates were both particle size and concentration dependent. Of note, high variation in some of the samples did prevent statistical significance being achieved at an α of ≤0.01 but was seen with an α of ≤0.05. Clot strength significantly increased from baseline controls in all groups except for 50 nm particles at 250 μg/ml (Figure 4). Interestingly, the rise in clot strength relative to control readouts was nearly uniform across all sample concentrations and particle sizes for cPS (Supplementary Figure 3). Only the highest concentration of the 500 nm cPS particles exhibited a slightly stronger effect on clot strength baseline deviation than the respective concentration of 50 or 100 nm particles. Representative TEG tracings for cPS can be seen in Figure 6.

### 3.3. Aminated polystyrene

Due to a lack of production of 50 nm aPS particles, only 100 and 500 nm aPS particles were tested. 100 nm aPS particles mirrored 100 nm cPS particle behavior on WB clotting dynamics (Figures 2–4). Clotting cascade activation times, time to initial fibrin polymerization, and rate of fibrin polymerization all significantly pointed towards quicker clotting dynamics in a concentration-dependent manner. Once again, the effects of Kaolin were surpassed in aPS containing samples at concentrations >25 μg/ml. Additionally, MA demonstrated a 12.6% rise above its respective control by the highest concentration of aPS 100 nm (69.4 ± 0.1 mm, *p*-value < 0.001). This rise in clot strength was marginally greater than both Kaolin samples (68.5 ± 1.1 mm) and the cPS 250 μg/ml group (67.0 ± 0.9 mm).

**Figure 7:**
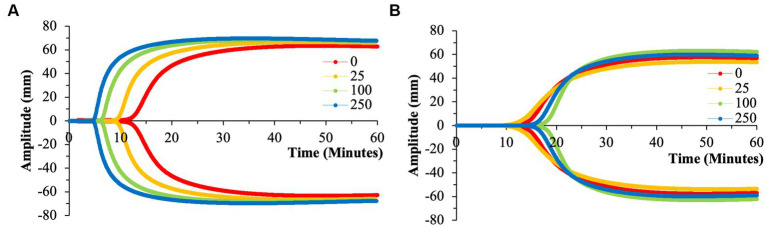
Representative TEG tracings for aPS with color-coded tracings based on the concentration of microparticles utilized. **(A)** 100  nm aPS. **(B)** 500  nm aPS. Minimal perturbation of clotting dynamics of the 500  nm microparticles compared to the effects of 100  nm particles. All tracings pictured were from a single channel (out of the quadruplicate) and are displayed as such for clarity purposes.

In contrast to aPS 100 nm particles, 500 nm aPS particle behavior was more aligned with 500 nm nPS particles. Changes to clotting dynamics were minimal across the spectrum of parameters collected (Figures 2–4). Trends towards quicker clotting cascade activation and initiation of fibrin deposition were present but did not achieve significance nor did they approximate the magnitude of effect seen with similar sized cPS particles. A 1.6% rise from baseline in MA was demonstrated with the highest concentration of 500 nm aPS particles, akin to the 1.4% rise seen in the comparable nPS group. Representative TEG tracings for aPS can be seen in Figure 7.

### 3.4. Impact of surface area

**Figure 8:**
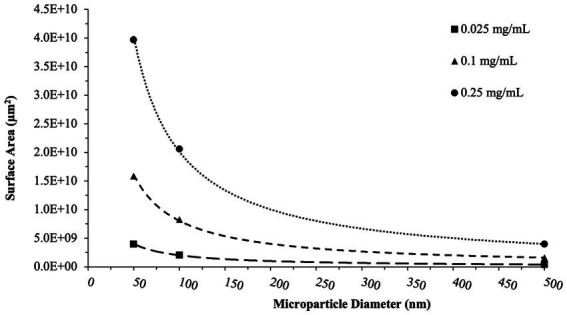
Total surface area calculations. These calculations reflect the surface area of nPS, cPS, or aPS whole blood was exposed to at a given concentration of microparticles. Trend lines capture the non-linear decrease in surface area with increasing particle size for a given concentration. aPS was not tested at the 50  nm size.

Analyses performed above viewed particle surface area and particle concentration as independent variables. However, in dealing with microparticles, total surface area is also an important aspect to consider. Utilizing particle size and concentration data from the particle manufacturer, we were able to perform simple calculations to generate estimated total particle surface areas within each experimental condition (Figure 8). Based on this data, the highest surface areas were noted in the 50 nm particle groups at 250 μg/ml (~4.0×10^10^ μm^2^), and the lowest surface area group naturally being the 500 nm particle groups at 25 μg/ml (~4.0×10^8^ μm^2^). Equally as important, the range of surface area was largest in the 50 nm groups, with a 100-fold change from the smallest to largest concentrations. As particle size increases, this range between concentrations becomes more subtle.

**Figure 9:**
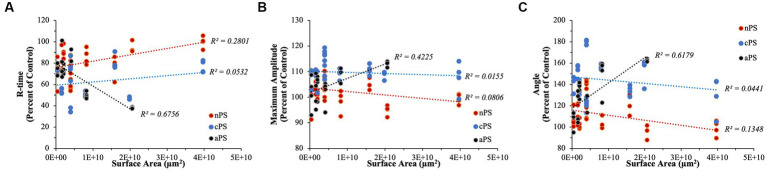
Linear regression analyses performed to explore any surface-area dependent effects that might exist on select TEG parameters. **(A)** R-time. **(B)** Maximum Amplitude. **(C)** Angle. aPS consistently demonstrated the highest R^2^ values in all of our analyses, indicating potential underlying importance of surface area on its pro-coagulable effects.

Accounting for the effect of surface area on the various perturbations of clotting dynamics, we begin to untangle which particles might be exhibiting effects in a surface area-dependent manner (Figure 9). Simple linear regression analyses were performed to explore any relationship between surface area and effects on clotting dynamics relative to a given day’s control. Both nPS and cPS demonstrated weak correlations with particle surface area when looking at speed of clotting cascade activation, rate of fibrin deposition, and maximal clot strength. On the other hand, aPS demonstrated the strongest surface-area dependent effect, with increasing surface area leading to faster clotting cascade activation (R^2^ = 0.6756), increasing maximal clot strength (R^2^ = 0.4225), and quicker fibrin deposition rates (R^2^ = 0.6179).

Performing simple linear regression, as above, can provide insightful information pertaining to relationships but, due to our experimental design with quadruplicates, it fails to properly account for variation within each set of replicates. To address this, we performed linear mixed model regression. As detailed above, surface area did not have a significant impact on determining R-time (*p*-value = 0.176), MA (*p*-value = 0.471), or Angle (*p*-value = 0.226) in the cPS group. With regards to nPS, both Angle (*p*-value = 0.028) and R-time (*p*-value <0.001) demonstrated having a significant impact by surface area, with increasing area leading to slower clotting dynamics – i.e., smaller Angles and longer R-times. Lastly, aPS demonstrated a highly dependent relationship of faster/stronger clot formation stemming from increasing surface areas – Angle (*p*-value < 0.001), MA (*p*-value < 0.001), and R-time (*p*-value < 0.001). The marginal R^2^ values (i.e., the variance explained by surface area) in our models were as follows: Angle – 0.511, MA – 0.321, R-time – 0.574.

## 4. Discussion

Utilizing human WB, we were able to explore the impact on coagulation presented by the introduction of polystyrene microplastics varying size, surface modification, and concentration. In employing TEG as our primary tool to capture clotting dynamics, we were able to study the effects in a WB system that approximates the complexity of *in-vivo* haemostasis/thrombosis – including all the cellular (ex. red blood cells, white blood cells, and platelets) and protein components. Studying coagulation in a relatively low-shear environment such as TEG allows us to focus in on the true effects microplastics have on coagulation without having to account for the intricacies that flow and endothelial cells might introduce into the system. Most importantly, in performing our experiments over the course of just a few days in a simple, systematic, and replicable fashion, we can truly explore the interplay of size and surface modification of PS microplastics in a manner that has yet to be done in the literature.

Across all the microplastics tested in our study, cPS most consistently induced a strong clotting response – regardless of size and/or concentration. Not only was it able to significantly reduce the speed of cascade initiation or fibrin deposition, cPS also allowed for the generation of clots with strengths at or above expected baselines. A lot of literature exists exploring the effect of cPS on coagulation, and the data tends to strongly vary depending on what system and what sizes are utilized. The consensus is that cPS operates via activation of the intrinsic pathway (akin to kaolin) by serving as a scaffold for factor XII, kallikrein, and HMWK to interact and generate activated factor XII (12, 15). These effects have been seen in both isolated protein systems in addition to plasma samples, and confirmed by targeted inhibition of activated factor XII with corn trypsin inhibitor (15). Oslakovic et al. found 220 nm cPS particles to be as effective as kaolin even at 50 μg/ml concentrations – roughly where we see a similar effect in the study presented here (15). More interestingly, cPS’ ability to serve as an activator of the intrinsic pathway is highly dependent on the geometries of the particles, particularly the surface curvature. Sanfins et al. and Oslakovic et al. discovered that at small particle sizes (26 and 24 nm), surface curvature became too high to promote proper complex formation (12, 15). On the other hand, particles of at least 60 nm in size were adequate to begin seeing intrinsic activation. In our study, we were able to begin seeing significant pro-coagulant effects starting at the 50 nm size cPS particles, although not to the effect of our larger cPS particles. In addition to cPS’ strong pro-coagulant effects, cPS has also been shown to promote strong platelet activation (18). The high MA readouts on TEG were thus expected findings as clot strength is largely due to platelet activity and their ability to forcefully contract on constructed fibrin scaffolds (19). Naturally, platelet activation and clotting cascade activation are intimately connected and feedback into one another. Thus, exact effects of cPS on these two pathways remains difficult to untangle with our experimental setup (20). Another possible explanation for cPS’ ability to generate clots with strengths above expected baselines/controls stems from the potentially increased rate of fibrin polymerization leading to stiffer clots. cPS’ negatively charged surface not only serves as an ideal activator of the intrinsic pathway but could also serve as a scaffold on which the prothrombinase complex (factors Xa, Va, and prothrombin) could effectively assemble and activate thrombin since individual components have an affinity for negatively charged surfaces (21). It is well documented in thrombosis literature that increasing amounts of thrombin promote the formation of thinner fibrin fibers that also impart more clot stability and strength (22, 23). Lastly, the tremendous surface area offered by microplastic cPS particles means that the impact of thrombin concentration gradients on network structure would be presumably minimized, thus ensuring greater homogeneity of fibrin microarchitecture (23).

Notably data does exist to also support cPS’ role as having anti-coagulant activity. *In-vivo* studies performed in a mouse model for thrombosis utilizing 60 nm cPS particles showed a propensity for cPS to decrease thrombus formation even though cPS could weakly promote small amounts of ADP-triggered platelet aggregation *in-vitro* (11, 24). Although not directly explored by Nemmar et al., the former effect was posited to be due to the negative surface charge of cPS potentially hindering platelet–platelet interactions stemming from repelling charges from phosphatidylserine on platelet surfaces. Data by Tran et al. illustrated that nPS (with its baseline slightly negative charge from sulfate esters) in fact decreased thrombin’s activity in converting fibrinogen to fibrin – in a purified thrombin/fibrinogen system - when nPS was pre-incubated with thrombin (10). Lastly, Griffin et al. also demonstrated, both via computational models and in *in-vitro* microfluidic WB experiments, that an optimal ratio of negatively charged nanoparticles to von Willebrand Factor (vWF) could significantly decrease thrombosis by stabilizing the globular form of vWF and thus minimizing its interactions with subendothelial collagen (13).

In contrast to cPS, nPS had an overall negligible effect on clotting dynamics regardless of particle size or concentration, especially in concentrations of ≥100 μg/ml and sizes of 100 and 500 nm. This overall minimal perturbation of clotting dynamics and thrombogenicity parallels a lot of the literature published on *in-vitro* and *in-vivo* studies (11, 18). Interestingly, our data quite reproducibly showed that nPS particles sized 50 nm with a concentration of 25 μg/ml significantly impacted speed of cascade activation, fibrin deposition rates, and even clot strength. These effects then seemed to taper off and become non-significant at larger sizes and higher concentrations of nPS. Given our experimental design, it is difficult to ascertain the exact mechanism for this reproducible effect – whether it be a result of platelet activation and/or interactions with the coagulation cascade. Of note, Smyth et al. demonstrated via *in-vitro* platelet aggregation assays that nPS can indeed trigger platelet aggregation when utilizing concentrations below 60 μg/ml, in contrast to prior studies that failed to demonstrate an nPS effect when utilizing concentrations ranging from 50 to 260 μg/ml (14). This effect did disappear in their *in-vivo* experiments. An alternative explanation for our data could be that the demonstrated slightly negative charge of nPS could be triggering activation of the intrinsic pathway akin to cPS, and this effect is simply lost at non-ideal concentrations or particle sizes (10, 25).

Through our work, we were additionally able to show that 100 nm aPS particles can affect clotting dynamics to the same extent, if not slightly more, than cPS particles. aPS particles promoted significant reductions in clotting time with concomitant rises in fibrin polymerization rates and clot strength all in a surface area-dependent manner. Intriguingly, all these effects are not observed with the 500 nm aPS particles, leading to a clotting phenotype nearly identical to nPS. Numerous studies have shown that aPS particles, and dendrimers with similar surface chemistries are able to increase rates of thrombosis both in *in-vivo* models in addition to being able to promote increased platelet aggregation and activation *in-vitro* (11, 14, 18, 24, 26, 27). An additional mechanism to possibly explain the effects of 100 nm aPS would be akin to cPS’ ability to activate the intrinsic pathway. In the case of aPS however, the extrinsic pathway would be implicated, as suggested by Sperling et al. (27). In their paper, Sperling et al. show that positively charged surfaces – polyethylenimine and poly-L-Lysine in their case – are able to strongly and specifically activate Factor VII Activating Protease, which in turn promotes activation of the extrinsic pathway in both plasma and whole blood experiments performed.

The 500 nm aPS’ inability to strongly promote clotting is quite an interesting finding with multiple possible explanations. In the case of cPS, particle size/geometry were clearly shown to impact the ability for the intrinsic pathway to be activated, and a similar phenomenon could be occurring in the case of the 500 nm aPS particle (12). Size-dependent effects on thrombin activation were also noted in silica nanoparticles (28). This possibility is less likely given that Sperling et al. utilized planar surfaces in their study, and the 500 nm aPS surface would be more akin to their conditions than a more curved 100 nm particle. Another potential explanation could be the impact of particle size on the protein corona of aPS 500 nm compared to aPS 100 nm. Particle size, and its surrounding medium, has been shown in multiple studies to impact the composition of the protein corona of nanoparticles in plasma (28–30). Lundqvist et al. demonstrated that the effect of size on protein corona composition is indeed more significant in aPS and cPS particles compared to nPS (29). As an example, the composition homology between the 50 nm and 100 nm cPS particles’ coronas approximated 50%. With this being said, a change in the protein binding of the 500 nm aPS particles could mean its potential affinity for some of the negatively charged coagulation factors – VII, IX, X – has increased, and thus impacting coagulation. Oslakovic et al. showed that aPS particles of 57 nm size largely reduced thrombin formation in plasma samples, due to their preferential binding of factors VII and IX (15). Interestingly, Chu et al. showed that soluble polyeythylenimine (a positively charged molecule) drastically impacted the ability for thrombin and fibrinogen to interact (31). These results are in direct contrast to Sperling et al. where polyethylenimine on a coated surface strongly activated coagulation, once again displaying the effect of particle geometry on coagulation. Lastly, a potential explanation for the inert presentation of 500 nm aPS could be that the particles aggregated, impacting their activity. Smyth et al. demonstrated baseline particle aggregation most drastically affecting nPS particles of size 50 and 100 nm (14). Additionally, Lundqvist et al. also experienced baseline particle aggregation in their 100 nm aPS group, although their particles differed from ours in that theirs were fluorophore labeled (29). Dynamic light scattering (DLS) experiments performed as part of prior work in our lab utilizing polystyrene microspheres from the same manufacturer demonstrated no particle aggregation; however, the use of whole blood clotting conditions in our present work make this an additional possibility to consider (10).

Identification of microplastics in human samples has been documented for a wide range of tissues including blood, liver, colon, and placental tissue (6, 32–34). Despite this, the effects of microplastic exposure on humans remains difficult to truly isolate and study, especially within the context of the cardiovascular system and thrombosis/hemostasis. As extensively discussed above, a lot of conclusions within the field stem from *in-vitro* studies, with few *in-vivo* studies, that often display a wide spectrum of effects stemming from microplastic exposure. It is well documented through animal studies that microplastics induce oxidative stress in numerous organs and promote a generally inflammatory response; however, little is known about their potential to affect human health from the perspective of altered thrombosis/hemostasis profiles *in-vivo* (35, 36). Significantly more work is required to unravel the intricate interplay of microplastics and human health from a coagulation point of view.

Limitations to our study include the limited sample size of healthy donors and the pristine/uniform nature of microplastics used that do not perfectly capture the diversity of microplastics that humans are exposed to. Given the number of replicates and conditions included in our study, it unfortunately was not possible to perform all experiments in a single day utilizing a single donor’s blood for all of the given particle sizes. Additionally, prior work in our lab has shown that storage of whole blood overnight does impact its clotting dynamics, thus we opted to split experiments over consecutive days with new healthy donors each day (16). Naturally, the above experimental design does subject our data to day-to-day clotting variations expected between healthy volunteers. With this in mind, these elements were taken into consideration throughout our data analysis/interpretation. While TEG is an invaluable clinical tool capable of displaying overall clotting dynamics and platelet activity, it does not provide great insight into the activity of discrete pathways, coagulation factors, platelet behavior, or clot microarchitecture as it is a more global clot analysis instrument. Equally as important, clotting within TEG occurs within a relatively static setting which contrasts physiologic clotting that often occurs in the presence of shear. Literature has demonstrated the notable impact of nanoparticles on the activation of the innate immune system with intricate pathways, such as the complement system and/or neutrophil extracellular trap formation – both of which can impact coagulation phenotypes and would ideally be accounted for (37, 38). With all of this being said, one of many future directions would include the study of microplastic impact on clotting dynamics and clot structure when conducted in the presence of shear as can be accomplished utilizing a Chandler Loop for example (39). Needless to say, the work presented herein serves as an important steppingstone in the understanding of microplastic impacts on human coagulation. The diversity and range of particles explored herein on a complete coagulation system utilizing a tool such as TEG provides useful insight for future research incorporating important variables, such as MP impact on clots formed under shear (40).

## Data availability statement

The original contributions presented in the study are included in the article/Supplementary material, further inquiries can be directed to the corresponding author.

## Ethics statement

The studies involving human participants were reviewed and approved by Indiana University School of Medicine (IRB protocol 1610652271). The patients/participants provided their written informed consent to participate in this study.

## Author contributions

AC performed experimental design, data collection, data analysis, data interpretation, and manuscript drafting. AH performed data collection and manuscript revision. NA performed experimental design, data analysis, data interpretation, and manuscript revision. All authors contributed to the article and approved the submitted version.

## Funding

This work was supported by the Department of Emergency Medicine at the Indiana University School of Medicine.

## Conflict of interest

The authors declare that the research was conducted in the absence of any commercial or financial relationships that could be construed as a potential conflict of interest.

## Publisher’s note

All claims expressed in this article are solely those of the authors and do not necessarily represent those of their affiliated organizations, or those of the publisher, the editors and the reviewers. Any product that may be evaluated in this article, or claim that may be made by its manufacturer, is not guaranteed or endorsed by the publisher.
