# Supplementary material for: Exploring microplastic impact on whole blood clotting dynamics utilizing thromboelastography
Source: Front Public Health. 2023 Jul 13;11:1215817. doi: 10.3389/fpubh.2023.1215817 (PMC10372794; doi:10.3389/fpubh.2023.1215817)

Supplemental Material

Exploring Microplastic Impact on Whole Blood Clotting Dynamics Utilizing Thromboelastography

Alexei Christodoulides[1], Abigail Hall[1] & Nathan J. Alves[1,2]*

[1]Indiana University School of Medicine – Department of Emergency Medicine, Indianapolis, IN, USA
[2]Purdue University – Weldon School of Biomedical Engineering, West Lafayette, IN, USA

***Correspondence:**
Nathan J. Alves, PhD
nalves@iu.edu

Supplementary Table 1
Summary statistics for nPS TEG parameters not discussed within the text.

Average

	K (min)	TMA (min)	G (d/sc)	E (d/sc)	TPI (/sec)	A30 (mm)	CLT (min)	CI
25 ug/mL 0.05u nPS	4.73	35.05	4447.88	88.95	9.43	-0.28	-32.48	-0.73
100 ug/mL 0.05u nPS	5.70	38.98	4044.63	80.88	7.28	-44.58	-20.48	-1.78
250 ug/mL 0.05u nPS	6.85	44.50	3575.55	71.50	5.23	-41.63	-16.33	-3.10

Standard Deviation

	K (min)	TMA (min)	G (d/sc)	E (d/sc)	TPI (/sec)	A30 (mm)	CLT (min)	CI
25 ug/mL 0.05u nPS	0.10	0.72	288.51	5.77	0.81	52.77	1.61	0.25
100 ug/mL 0.05u nPS	0.73	1.47	255.86	5.12	1.28	1.58	1.62	0.41
250 ug/mL 0.05u nPS	0.34	1.44	123.53	2.45	0.32	0.62	1.84	0.36

Average

	K (min)	TMA (min)	G (d/sc)	E (d/sc)	TPI (/sec)	A30 (mm)	CLT (min)	CI
25 ug/mL 0.1u nPS	3.30	36.05	8214.63	164.30	24.90	-61.95	-24.55	1.40
100 ug/mL 0.1u nPS	3.25	37.10	7747.20	154.95	25.05	-60.55	-29.98	1.23
250 ug/mL 0.1u nPS	3.90	39.68	7049.25	141.00	18.28	-58.45	-19.83	0.83

Standard Deviation

	K (min)	TMA (min)	G (d/sc)	E (d/sc)	TPI (/sec)	A30 (mm)	CLT (min)	CI
25 ug/mL 0.1u nPS	0.22	1.60	257.32	5.12	2.08	0.53	1.56	0.26
100 ug/mL 0.1u nPS	0.58	0.96	828.97	16.57	6.77	2.67	0.85	0.59
250 ug/mL 0.1u nPS	0.64	1.59	339.77	6.79	2.53	1.24	1.39	0.46

Average

	K (min)	TMA (min)	G (d/sc)	E (d/sc)	TPI (/sec)	A30 (mm)	CLT (min)	CI
25 ug/mL 0.5u nPS	4.13	38.18	6966.18	139.35	17.33	-57.85	-21.10	0.53
100 ug/mL 0.5u nPS	3.75	38.68	7157.35	143.18	19.13	58.25	-60.93	0.30
250 ug/mL 0.5u nPS	3.70	37.50	6818.53	136.40	18.50	-57.15	-24.40	0.18

Standard Deviation

	K (min)	TMA (min)	G (d/sc)	E (d/sc)	TPI (/sec)	A30 (mm)	CLT (min)	CI
25 ug/mL 0.5u nPS	0.50	3.37	1262.10	25.25	5.27	4.11	3.83	1.08
100 ug/mL 0.5u nPS	0.39	2.28	346.45	6.93	1.97	1.18	0.40	0.47
250 ug/mL 0.5u nPS	0.28	1.40	394.00	7.87	2.32	1.33	8.90	0.28

Supplementary Table 2
Summary statistics for cPS TEG parameters not discussed within the text.

Average

	K (min)	TMA (min)	G (d/sc)	E (d/sc)	TPI (/sec)	A30 (mm)	CLT (min)	CI
25 ug/mL 0.05u cPS	5.38	37.25	4502.55	90.05	8.35	-47.50	-14.48	-1.28
100 ug/mL 0.05u cPS	4.78	38.53	4330.53	86.60	9.13	-46.58	-20.73	-1.73
250 ug/mL 0.05u cPS	5.03	40.78	4126.80	82.53	8.45	45.13	-40.10	-1.75

Standard Deviation

	K (min)	TMA (min)	G (d/sc)	E (d/sc)	TPI (/sec)	A30 (mm)	CLT (min)	CI
25 ug/mL 0.05u cPS	0.35	2.16	467.37	9.37	0.81	2.54	2.60	0.54
100 ug/mL 0.05u cPS	0.30	0.95	148.26	2.96	0.65	0.98	1.07	0.34
250 ug/mL 0.05u cPS	0.95	1.98	425.75	8.53	2.26	2.60	2.19	0.37

Average

	K (min)	TMA (min)	G (d/sc)	E (d/sc)	TPI (/sec)	A30 (mm)	CLT (min)	CI
25 ug/mL 0.1u cPS	1.70	30.75	9637.60	192.78	57.18	-34.53	-30.28	2.18
100 ug/mL 0.1u cPS	1.18	27.70	10222.03	204.45	84.98	67.13	-35.95	2.83
250 ug/mL 0.1u cPS	1.13	27.48	10142.98	202.88	92.08	66.95	-35.83	2.90

Standard Deviation

	K (min)	TMA (min)	G (d/sc)	E (d/sc)	TPI (/sec)	A30 (mm)	CLT (min)	CI
25 ug/mL 0.1u cPS	0.18	0.52	910.43	18.23	9.75	64.76	1.53	0.37
100 ug/mL 0.1u cPS	0.05	1.15	487.13	9.73	7.99	1.11	1.57	0.24
250 ug/mL 0.1u cPS	0.05	1.29	408.92	8.17	6.88	0.91	1.21	0.08

Average

	K (min)	TMA (min)	G (d/sc)	E (d/sc)	TPI (/sec)	A30 (mm)	CLT (min)	CI
25 ug/mL 0.5u cPS	2.43	34.98	7689.78	153.80	32.20	-60.65	-24.65	0.85
100 ug/mL 0.5u cPS	2.05	29.83	8236.90	164.73	40.50	-61.98	-29.18	1.60
250 ug/mL 0.5u cPS	1.25	24.23	9511.25	190.20	75.85	64.65	-35.48	2.78

Standard Deviation

	K (min)	TMA (min)	G (d/sc)	E (d/sc)	TPI (/sec)	A30 (mm)	CLT (min)	CI
25 ug/mL 0.5u cPS	0.30	0.79	731.38	14.65	6.95	2.15	0.78	0.19
100 ug/mL 0.5u cPS	0.17	0.56	248.58	5.00	3.36	0.88	0.46	0.18
250 ug/mL 0.5u cPS	0.13	0.40	569.36	11.39	12.35	1.26	1.49	0.13

Supplementary Table 3
Summary statistics for aPS TEG parameters not discussed within the text.

Average

	K (min)	TMA (min)	G (d/sc)	E (d/sc)	TPI (/sec)	A30 (mm)	CLT (min)	CI
25 ug/mL 0.1u aPS	1.90	30.95	9160.25	183.20	49.48	-64.80	-27.33	1.93
100 ug/mL 0.1u aPS	1.23	25.95	10265.75	205.33	85.53	67.03	-32.88	2.95
250 ug/mL 0.1u aPS	1.00	23.75	11309.80	226.23	113.38	69.00	-35.83	3.50

Standard Deviation

	K (min)	TMA (min)	G (d/sc)	E (d/sc)	TPI (/sec)	A30 (mm)	CLT (min)	CI
25 ug/mL 0.1u aPS	0.24	0.79	1019.69	20.37	10.82	2.61	1.07	0.41
100 ug/mL 0.1u aPS	0.05	0.73	712.13	14.22	10.95	1.52	1.17	0.39
250 ug/mL 0.1u aPS	0.08	0.73	395.89	7.92	6.35	0.88	0.68	0.12

Average

	K (min)	TMA (min)	G (d/sc)	E (d/sc)	TPI (/sec)	A30 (mm)	CLT (min)	CI
25 ug/mL 0.5u aPS	4.18	36.90	6920.68	138.40	16.95	-57.68	-23.85	0.53
100 ug/mL 0.5u aPS	3.58	36.98	6940.68	138.85	20.55	-57.58	-25.65	0.15
250 ug/mL 0.5u aPS	3.28	36.98	6894.73	137.90	21.48	-57.15	-26.10	-1.83

Standard Deviation

	K (min)	TMA (min)	G (d/sc)	E (d/sc)	TPI (/sec)	A30 (mm)	CLT (min)	CI
25 ug/mL 0.5u aPS	0.62	1.54	1040.76	20.82	4.12	3.81	1.59	0.38
100 ug/mL 0.5u aPS	0.78	0.67	1035.71	20.69	7.04	3.72	0.44	0.24
250 ug/mL 0.5u aPS	0.36	1.04	774.16	15.51	4.35	2.92	1.28	3.42

Supplementary Figure 1

TEG parameters collected from platelet mapping assays performed on our three volunteer blood donors. Note, assays depicted here did not involve any experimental manipulation (i.e., inclusion of microplastics).

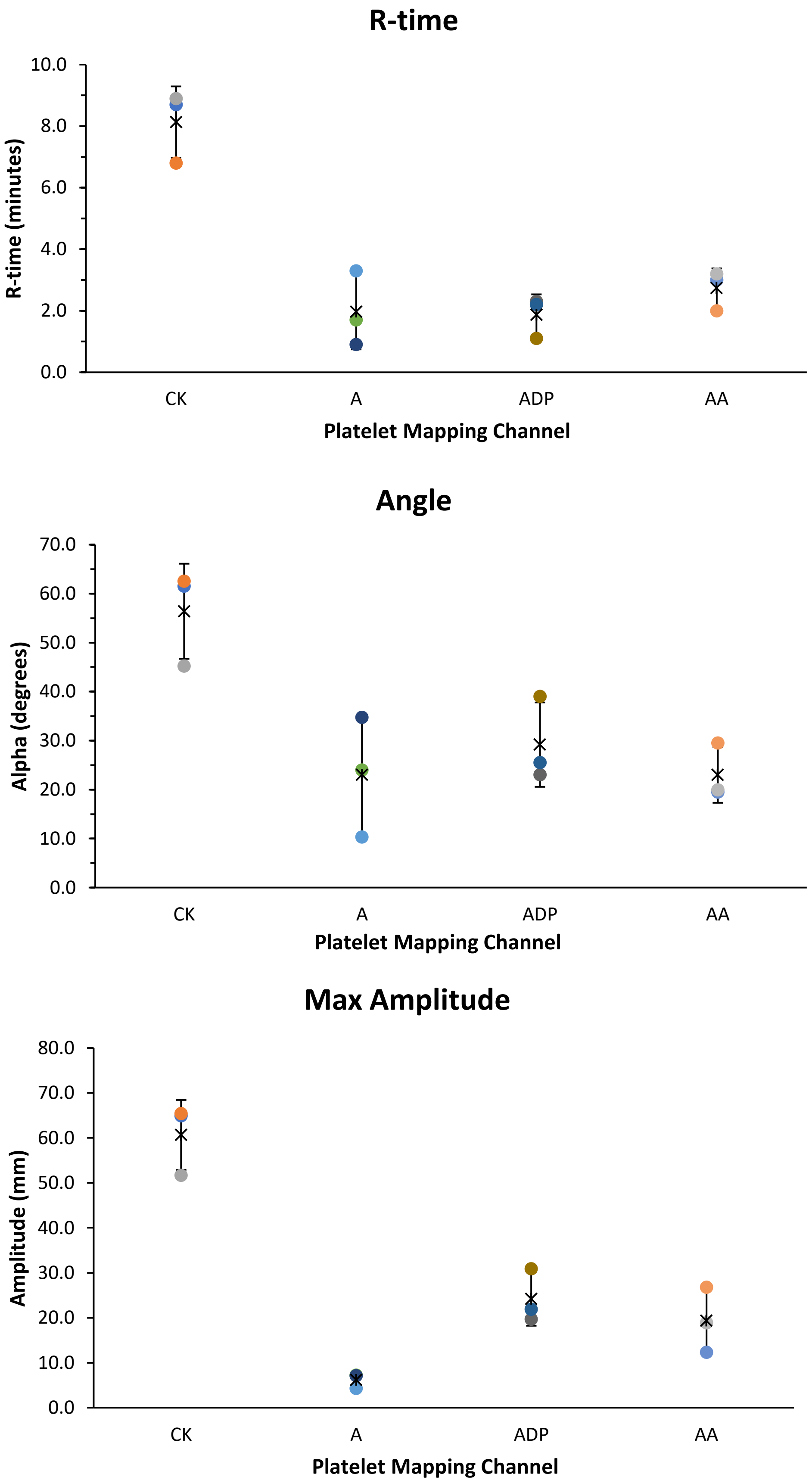

Supplementary Figure 2

Statistical comparisons based on nPS particle size within a particular concentration utilized. Note, data presented here is represented as a percentage of baseline controls to account for normal variability existing between healthy blood donors. Asterisks denote level of significance between respective groups: (*) P-value ≤0.05, (**) P-value ≤0.01, (***) P-value≤0.001.

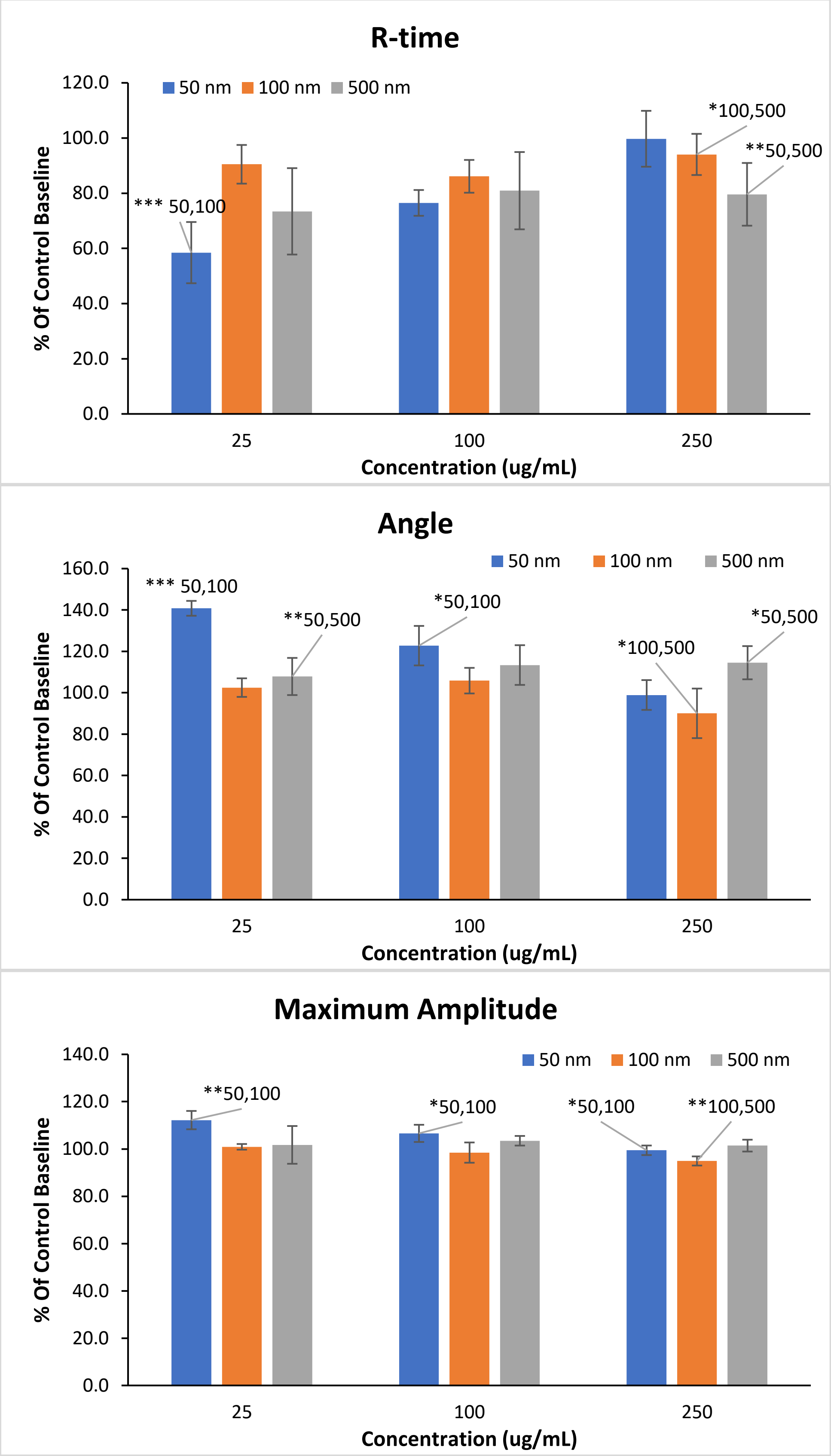

Supplementary Figure 3
Statistical comparisons based on cPS particle size within a particular concentration utilized. Note, data presented here is represented as a percentage of baseline controls to account for normal variability existing between healthy blood donors. Asterisks denote level of significance between respective groups: (*) P-value ≤0.05, (**) P-value ≤0.01, (***) P-value≤0.001.

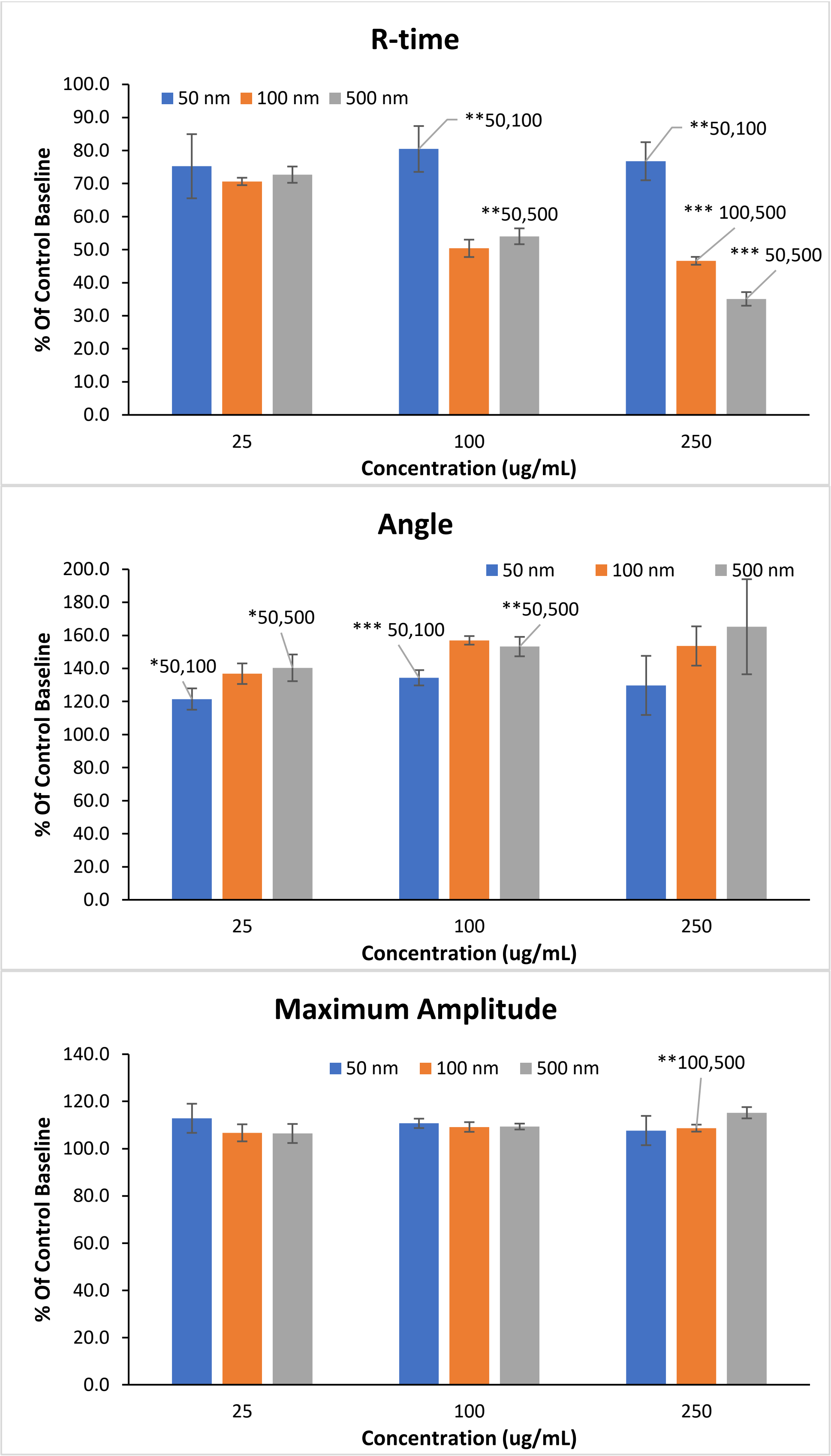

Supplementary Figure 4
Statistical comparisons based on aPS particle size within a particular concentration utilized. Note, data presented here is represented as a percentage of baseline controls to account for normal variability existing between healthy blood donors. Asterisks denote level of significance between respective groups: (*) P-value ≤0.05, (**) P-value ≤0.01, (***) P-value≤0.001.

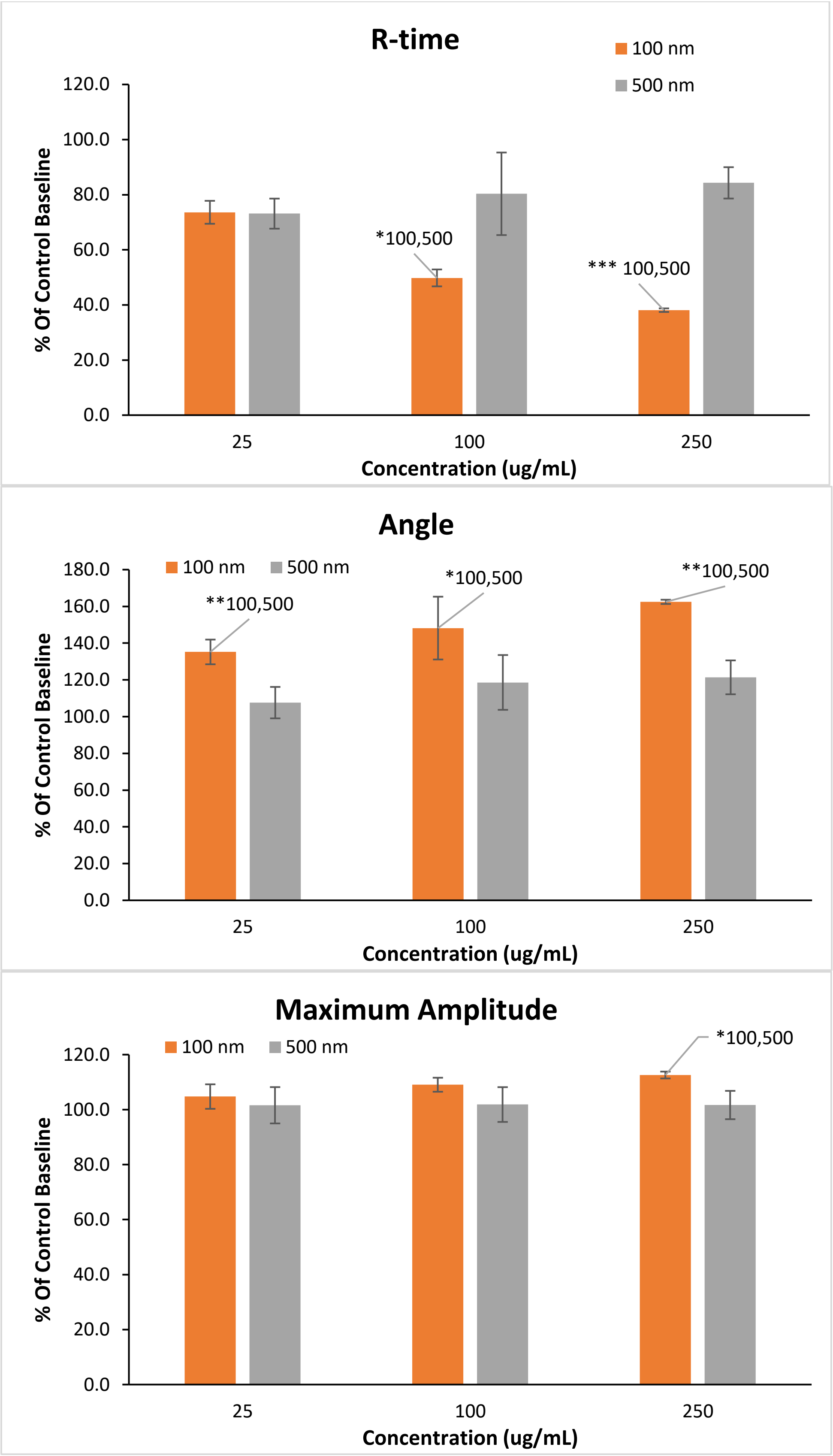

Supplement: Supplementary file 1 [file Data_Sheet_1.PDF]
